# Mechanical Thrombectomy of COVID-19 positive acute ischemic stroke patient: a case report and call for preparedness

**DOI:** 10.1186/s12883-020-01930-x

**Published:** 2020-09-24

**Authors:** Ossama Yassin Mansour, Amer M. Malik, Italo Linfante

**Affiliations:** 1 Neurology Department, Stroke and Neurointerventional Unit, Alexandria University, Faculty of Medicine, Alexandria, Egypt; 2 Vascular Neurology Division, Department of Neurology, University of Miami Miller School of Medicine, Miami, USA; 3 Miami Cardiac and Vascular Institute, Baptist Neuroscience Institute, Florida International University, Miami, USA

**Keywords:** Acute ischemic stroke, Mechanical thrombectomy, COVID-19, Coronavirus, SARS-CoV-2, Stroke workflow, Personal protective equipment, Large vessel occlusion

## Abstract

**Background:**

The novel coronavirus (COVID-19) global pandemic is associated with an increased incidence of acute ischemic stroke (AIS) secondary to large vessel occlusion (LVO). The treatment of these patients poses unique and significant challenges to health care providers requiring changes in existing protocols.

**Case presentation:**

A 54-year-old COVID-19 positive patient developed sudden onset left hemiparesis secondary to an acute right middle cerebral artery occlusion (National Institutes of Health Stroke Scale (NIHSS) score = 11). Mechanical thrombectomy (MT) was performed under a new protocol specifically designed to maximize protective measures for the team involved in the care of the patient. Mechanical Thrombectomy was performed successfully under general anesthesia resulting in TICI 3 recanalization. With regards to time metrics, time from door to reperfusion was 60 mins. The 24-h NIHSS score decreased to 2. Patient was discharged after 19 days after improvement of her pulmonary status with modified Rankin Scale = 1.

**Conclusion:**

Patients infected by COVID-19 can develop LVO that is multifactorial in etiology. Mechanical thrombectomy in a COVID-19 confirmed patient presenting with AIS due to LVO is feasible with current mechanical thrombectomy devices. A change in stroke workflow and protocols is now necessary in order to deliver the appropriate life-saving therapy for COVID-19 positive patients while protecting medical providers.

## Background

The first case of SARS-CoV-2 infection in Wuhan Province of China in December 2019 [1]. As of 29 Feb. 2020, the number of laboratory-confirmed COVID-19 cases has exceeded 500,000 cases globally [2]. The clinical presentation of COVID-19 patients varies considerably, ranging from asymptomatic infection to severe pneumonia that may lead to respiratory failure and death [3]. Recently, Li et al. performed a meta-analysis of six studies reporting data on COVID-19 patients. The authors reported the presence of cardiac and cerebrovascular diseases in16.4% of this population. In addition, they reported that the incidence of vascular disease was three-fold higher among severe COVID-19 patients requiring ICU care [4] In addition, Li YaB et al. reported that 13 of 221 (5.8%) of the cases who suffered from a SARS-CoV-2 infection developed acute cerebrovascular disease (CVD), possibly related to the prothrombotic effect of the host inflammatory response to the virus. Older patients with comorbidities such as diabetes, pulmonary diseases and hypertension are more likely to develop acute CVD. In addition, the development of CVD in these patients is a significant negative prognostic factor for survival and recovery from COVID-19 infections [5]. Therefore, acute CVD is not uncommon in COVID-19 and is a significant risk factor for mortality in these patients.

Multiple controlled randomized clinical trials have demonstrated overwhelming superiority of mechanical thrombectomy (MT) compared to medical management in AIS secondary to large vessel occlusion (LVO) [6]. However, performing MT in patient with COVID 19 poses significant health risk in health care providers and requires changes in existing protocols. We report a clinical case of a COVID-19 positive patient presenting to our center with AIS secondary to LVO, treated successfully by MT. We also describe the possible modifications for measures and workflow to deliver appropriate treatment for such patients in the era of the COVID-19 global pandemic.

## Case presentation

**Fig. 1:**
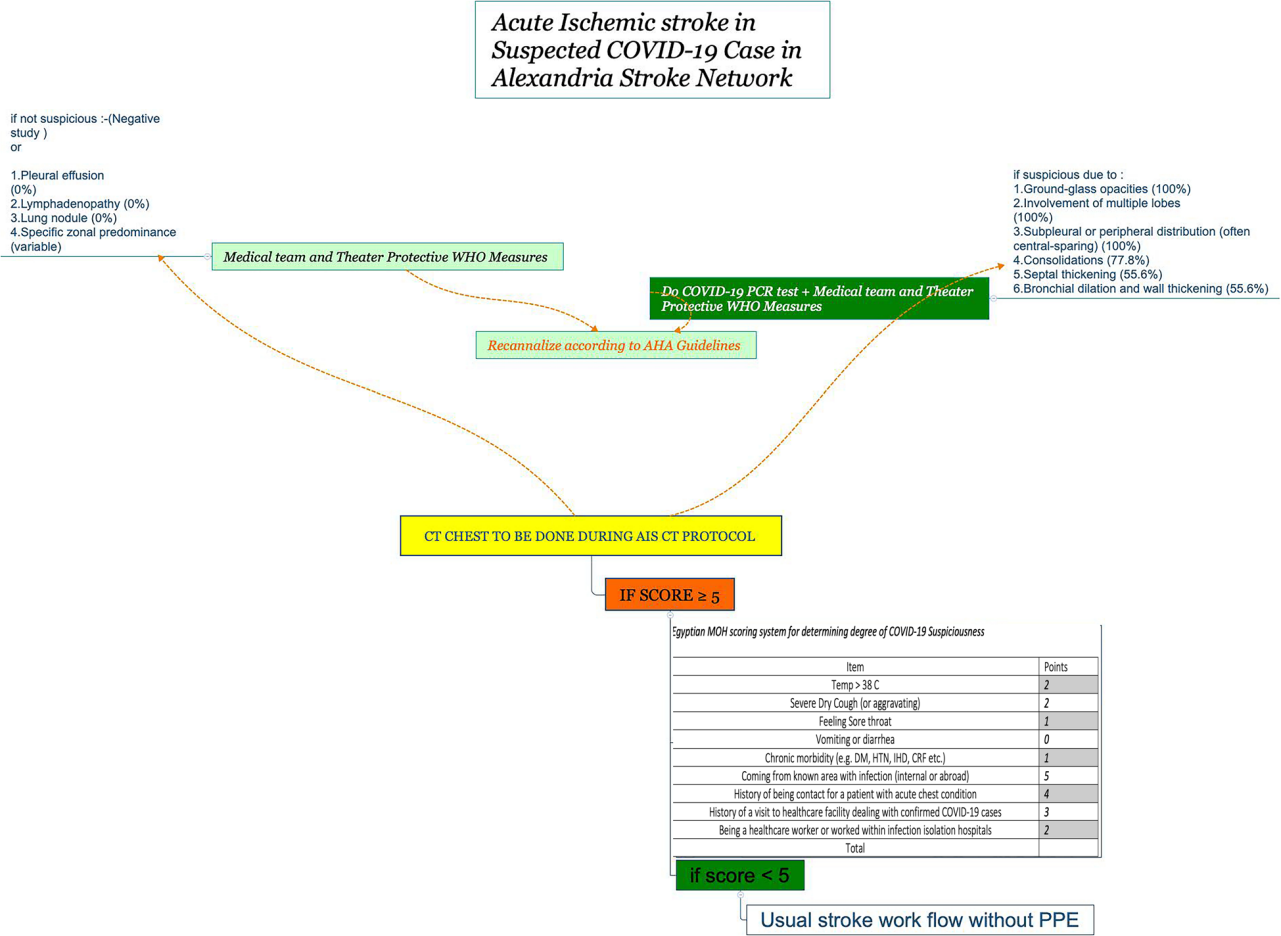
Proposed modified stroke protocol based on Egyptian MOH score system^16^ for suspecting patient for COVID-19

A 54-year-old Italian patient was diagnosed and confirmed to be COVID-19 positive by *Real Time* quantitative Reverse transcription polymerase chain reaction *RT-qPCR* test performed with throat swabs. Six days after the diagnosis, she developed sudden onset of left hemiparesis and neglect. Patient was transferred emergently via ambulance from a COVID-19 isolation hospital to Alexandria University Affiliated Hospital where patient arrived approximately 4.5 h after symptom onset. Continuous communication with the ambulance team occurred, including recommendation for intubation of the patient in order to minimize in-hospital droplet and contact precautions to shorten door-to-needle time. The patient’s oxygen saturation subsequently dropped below 90% in the ambulance and intubation was performed emergently by ambulance team. Upon hospital arrival, she was admitted via an isolated and guarded entrance specifically designed for: i) patients with symptoms suspicious for COVID-19 and/or; ii) diagnosed as being COVID-19 positive as per our workflow AIS protocol (Fig. 1). In the emergency room, evaluation indicated that the patient’s history was negative for atrial fibrillation, hypertension, or diabetes. Neurological examination revealed left lower facial palsy, hemiparesis, hemisensory deficit, and right gaze deviation. The National Institutes of Health Stroke Scale (NIHSS) = 11.

**Fig. 2:**
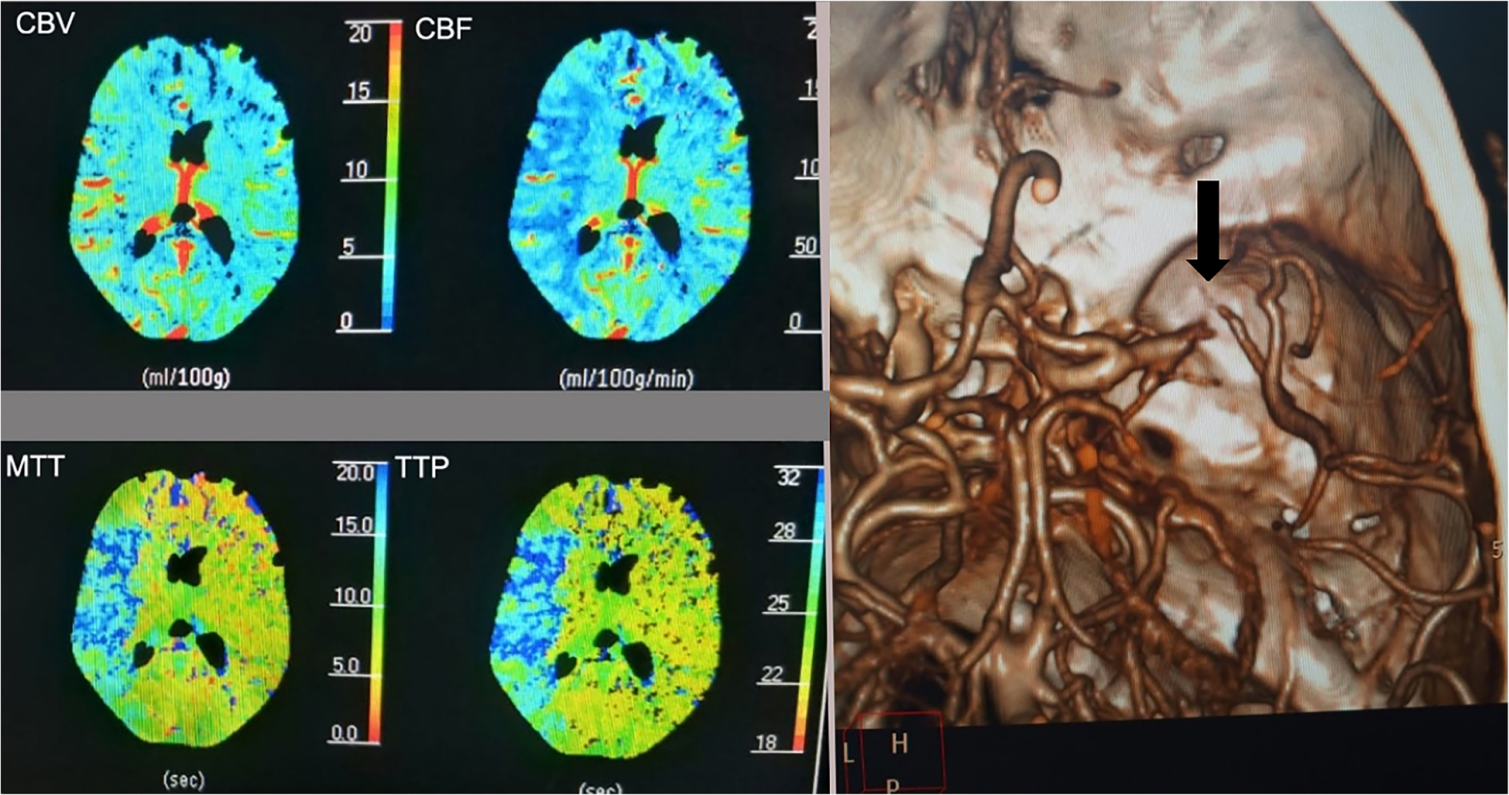
CTP & CTA of the patient showing acute right M1 occlusion (arrow) with large area of ischemic penumbra

At admission, computed tomographic angiography (CTA) and perfusion (CTP) imaging revealed acute right MCA M1 occlusion with a large area of ischemic penumbra (Fig. 2). Admission ABG and blood sample lab tests are summarized in Table 1. Due to 4.5-h time delay from symptom onset to presentation to our ED, the patient was excluded to receive intravenous tissue plasminogen activator (IV tPA) according to current guidelines. Written informed consent for mechanical thrombectomy (MT) was obtained from the patient’s family through video conference (according to hospital protocol for consent taking for patients with AIS). The endovascular procedure was performed with a transfemoral approach under general anesthesia (GA).

**Table 1:** Laboratory investigation on admission of the COVID-19 positive patient presenting with acute ischemic stroke

Test	Vule at admission
**ABG at admission**	
**pH**	7.049
**PCO2**	76.4 mmHg
**PO2**	66.2 mmHg
**SaO2**	91.8%.
**Blood samples**	
**ALT**	97 U/L
**creatinine**	157 mol/L
**D-dimer**	490 μg/L
**Platelet count, 10**^**9**^**/L**	190
**WBC, 10**^**9**^**/L**	8
**Lymphocyte count, 10**^**9**^**/L**	0.4
**CRP**	21
**PCT**	12

*ABG* arterial blood gas, *ALT* alanine aminotransferase, *CRP* c-reactive protein, *PCT* procalcitonin, *PCO2* partial pressure of carbon dioxide, *PO2* partial pressure of oxygen, *SaO2* oxygen saturation, *WBC* white blood count

**Fig. 3:**
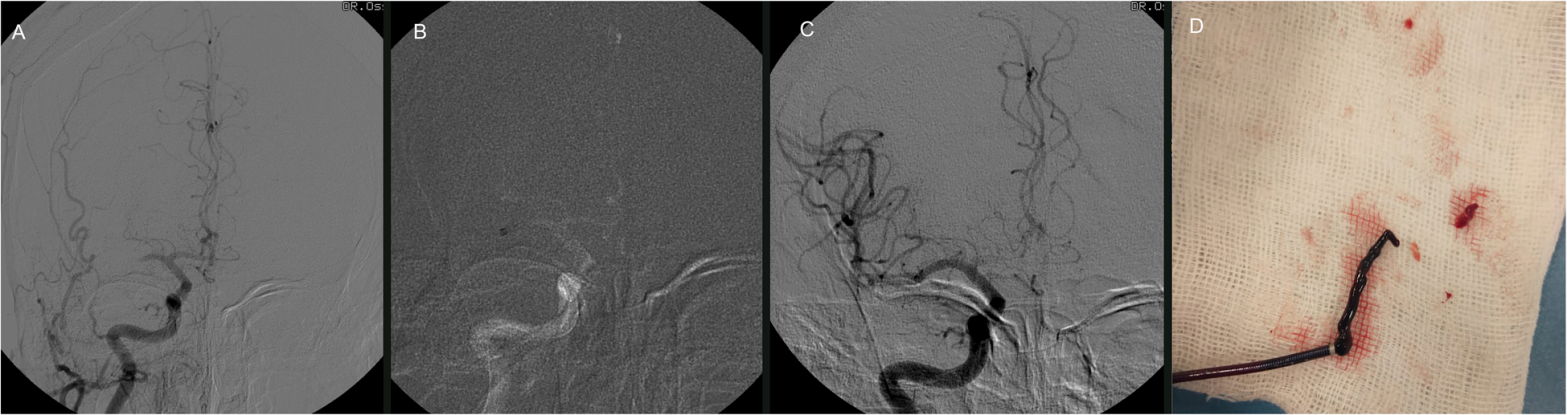
**a**: Digital subtraction angiography showing right M1 occlusion. **b**: Reperfusion catheter navigated to occlusion site. **c** First pass TICI 3 recanalization. **d** Thrombus at the tip of the reperfusion catheter

The neuroendovascular team prepared the patient for the procedure, under a pre-determined special personal protective equipment (PPE) protocol for the care of COVID-19 patients. Cerebral angiography revealed occlusion of the right MCA (Fig. 3a). Mechanical thrombectomy was performed according to current protocols. Briefly, after obtaining access via the right femoral artery, an 8-Fr guiding catheter (Guider softip XF; Stryker Neurovascular, Fremont, CA, USA) was advanced into the right internal carotid artery. A 6-Fr intermediate catheter (CAT™ 6 Distal Access Catheter; Stryker Neurovascular, Fremont, CA, USA) was navigated over a micro guidewire into the occlusion site in the MCA (Fig. 3b). A direct aspiration first pass technique (ADAPT) technique was performed via the CAT 6 resulting in first-pass successful recanalization with Thrombolysis in Cerebral Infarction (TICI) grade 3 recanalization. Symptom onset to reperfusion was 330 min, door to reperfusion time was 60 min and groin-to-reperfusion time was 22 min (Fig. 3c). The thrombus extracted was sent for histological examination (Fig. 3d). No procedure-related complications occurred. After procedure completion, the patient was transferred back to the specialized COVID-19 hospital and managed in the ICU. The patient’s left hemiparesis improved the day after the MT procedure. After extubation, her NIHSS score was = 2.

### Mechanical Thrombectomy personal protective measures

Per our protocol for the care of COVID-19 patients, she was transported to the angiography suite already intubated to avoid exposure to the staff. During the procedure the number of healthcare workers in the angiography suite was minimized to operator, nurse, and a single anesthesiology attending. Medical staff members received their personal protective equipment (PPE) first. The PPE was packed in a special box labeled ‘COVID-19’ and included the following: yellow gown, gloves, visor, mask, surgical hoods, and scrubs. Removal of all personal equipment before entering the angiography suite was mandatory.

Briefly, the protocol started by wearing boots by slipping the feet into them without touching before proceeding to the scrubbing area. The next step involved putting on protective leads that are classified as “dirty” and restored for such cases. Next hand washing was followed by placement of PPE head gear which included a surgical hood followed by cap, mask, and visor. Placement of this PPE was followed by placement of an N95 mask. Again, hands were washed before putting on the yellow gown with the help of another member in the angiography suite. For operators only, the next step was to scrub as they would normally do, except they completed this task with gloves and yellow gown on. For non-operators, they wore the gloves included in the COVID-19 box but they did not wear sterile gloves. Once all of the PPE was on, the team proceeded to the angiography suite for the intervention.

After the procedure was concluded, the patient was safely transferred by ambulance to the original transferring hospital for further management. At this point, members of the team began to take off their respective PPE. The visor was removed first due to potential contamination with blood or other fluids and disposed of in a specially labeled bin. Subsequently, the sterile gloves were taken off and disposed of in the same bin. The gown was removed by tearing the ties and taking it off by rolling on the inside and the gown was disposed of in the bin. The last pair of gloves were removed and disposed of in the bin. Then bin trash and its contents were treated according the regulations recommended by local health authority.

## Discussion and conclusion

This is a report of a COVID 19 patient with AIS secondary due to LVO that underwent successful MT with excellent clinical and angiographic outcome in contrary to majority of reported case prognosis [7]. Patients with COVID-19 infection, and in particular those requiring ICU care, are more at risk to develop CVD and stroke^4^. The acute management of COVID-19 patients with a stroke is challenging because it requires specific protocols to minimize exposure to the operators and staff involved in the care of the patient in the angiography suite. Current CDC guidelines have been previously published and were followed in this case. Mechanical thrombectomy is standard of care for AIS secondary to LVO [8, 9]. Modification of the mechanical thrombectomy workflow of LVO AIS patients should be considered to ensure the safety of the medical team and the ongoing availability of the service with readiness of the receiving hospital to handle patients during such difficult times without disturbing existing pathways.

Another group of patients that could be included in such protocols are those patients under investigation (PUI) who are not yet confirmed to be COVID-19 positive status and still they are potentially infectious [10]. In this group of PUI patients who present with AIS, some protective measures during the pandemic time should not waste the precious time necessary to receive AIS reperfusion therapy. This goal relies on the importance of having a protocol to deal with both subgroups of AIS in relation to COVID-19 infection (Fig. 1).

There is little data regarding acute CVD following COVID-19 infection [5]. Inflammation has been increasingly recognized as a key contributor to the pathophysiology of cerebrovascular diseases [11]. In particular in LVO, the inflammatory response may trigger events in the coagulation cascade intravascularly [12, 13]. This seems to be the case in our patients. In the patient described in this report, there may have been increase in the inflammatory response (elevated C-reactive protein and PCT in Table 1) and abnormal blood coagulation function (elevated D-dimer in Table 1) in the early stage which could be one of the main reasons of acute onset CVD cited in a prior report [5]. In addition, since respiratory infections and A-V mismatch occur this may affect blood oxygenation putting penumbra at risk, so these patients need aggressive medical management of their underlying infection.

No defined data can confirm a causal relationship between SARS-CoV-2 and ischemic stroke, since competing vascular risk factors and mechanisms were present in most of the reported patients. However, some reports suggested that COVID-19 might stimulate the production of antiphospholipid antibodies (aPL) [14] as a mechanism of ischemic stroke, although postinfection aPL are usually transient and unassociated with thrombosis. Nevertheless, our case suggests that stroke linked to Covid-19 infection can occur in the context of a systemic highly prothrombotic state, as no other vascular risk was report in the current case.

With regards to changes in protocols and workflow, undoubtedly COVID-19 frontline healthcare workers are at a high risk for infection. Data from China’s National Health Commission shows that more than 3300 healthcare workers have been infected as of early March 2020 and by the end of February at least 22 individuals had died. In Italy, 20% of responding healthcare workers were infected, and some have died. Reports from medical staff describe physical and mental exhaustion, the torment of difficulty with triage decisions, and the pain of losing patients and colleagues, all in addition to the active infection risk [15]. Therefore, the proper use of PPE and following recommended protocols are key to guaranteeing the safety of healthcare workers and are essential to run any acute treatment workflow like that employed for AIS [16].

Intubating such patients in the angiography suite could lead to increasing the exposure to the medical team in the stroke facility. Different protocols could be implemented to decrease such exposure in our case. We recommend intubating the patient before transfer to treating hospital or angiography suite in negative pressure-controlled room in ED or ICU that already had all PPE needed to deal with such cases following COVID-19 protocols. Other protocols for decreasing exposure during intubation may be used like intubating in a negative pressure emergency department room or under cover with video glidescope to avoid direct exposure to the patient’s droplets. Additionally, the use of telemedicine in diagnosis and administration of IV thrombolysis may provide the ‘social distancing’ in stroke practice that could decrease the infection exposure of healthcare workers and should be considered in an AIS management protocol modified to accommodate the recent COVID-19 situation worldwide. Mechanical thrombectomy was done under GA. Caution should be taken as these patients are more prone to acute respiratory distress syndrome (ARDS) and recommendations for hemodynamics, pulmonary function, and anesthesia drug selection should be considered as in published guidelines. Patients with COVID-19 infection are at increased risk of cerebrovascular diseases and acute ischemic stroke. Mechanical thrombectomy in AIS secondary to LVO in COVID-19 patients is feasible. However, existing stroke protocols should be modified following CDC guidelines for PUI or confirmed COVID-19 cases to deliver effective care for these patients and protect healthcare workers in this field.

## Data Availability

Not applicable.

## Abbreviations

AIS: Acute ischemic stroke
ADAPT: A direct aspiration first pass technique
CTA: Computed tomographic angiography
CTP: Computed tomographic perfusion
CVD: Cerebrovascular disease
GA: General anesthesia
IV tPA: Intravenous tissue plasminogen activator
MCA: Middle cerebral artery
MT: Mechanical thrombectomy
NIHSS: National Institutes of Health Stroke Scale
TICI: Thrombolysis in Cerebral Infarction
